# Spontaneous γH2AX Foci in Human Solid Tumor-Derived Cell Lines in Relation to p21^WAF1^ and WIP1 Expression

**DOI:** 10.3390/ijms160511609

**Published:** 2015-05-20

**Authors:** Razmik Mirzayans, Bonnie Andrais, April Scott, Ying W. Wang, Robert H. Weiss, David Murray

**Affiliations:** 1 Department of Oncology, University of Alberta, Cross Cancer Institute, Edmonton, AB T6G 1Z2, Canada; E-Mails: bonnie.andrais@albertahealthservices.ca (B.A.); april.scott@albertahealthservices.ca (A.S.); ywwang@ualberta.ca (Y.W.W.); david.murray5@albertahealthservices.ca (D.M.); 2 Division of Nephrology, Department of Internal Medicine, University of California, Davis, CA 95616, USA; E-Mail: rhweiss@ucdavis.edu; 3 Department of Medicine, Mather VA Medical Center, Sacramento, CA 95655, USA

**Keywords:** p21^WAF1^, WIP1, γH2AX foci, p53, apoptosis

## Abstract

Phosphorylation of H2AX on Ser139 (γH2AX) after exposure to ionizing radiation produces nuclear foci that are detectable by immunofluorescence microscopy. These so-called γH2AX foci have been adopted as quantitative markers for DNA double-strand breaks. High numbers of spontaneous γH2AX foci have also been reported for some human solid tumor-derived cell lines, but the molecular mechanism(s) for this response remains elusive. Here we show that cancer cells (e.g., HCT116; MCF7) that constitutively express detectable levels of p21^WAF1^ (p21) exhibit low numbers of γH2AX foci (<3/nucleus), whereas p21 knockout cells (HCT116p21−/−) and constitutively low p21-expressing cells (e.g., MDA-MB-231) exhibit high numbers of foci (e.g., >50/nucleus), and that these foci are not associated with apoptosis. The majority (>95%) of cells within HCT116p21−/− and MDA-MB-231 cultures contain high levels of phosphorylated p53, which is localized in the nucleus. We further show an inverse relationship between γH2AX foci and nuclear accumulation of WIP1, an oncogenic phosphatase. Our studies suggest that: (i) p21 deficiency might provide a selective pressure for the emergence of apoptosis-resistant progeny exhibiting genomic instability, manifested as spontaneous γH2AX foci coupled with phosphorylation and nuclear accumulation of p53; and (ii) p21 might contribute to positive regulation of WIP1, resulting in dephosphorylation of γH2AX.

## 1. Introduction

Phosphorylation of the histone H2A variant H2AX on Ser139 is an important event in the cellular response to genotoxic agents that induce DNA double-strand breaks (DSBs) [1]. This phosphorylation can be mediated by different protein kinases, including ataxia telangiectasia mutated (ATM) and other members of the phosphatidylinositol 3-kinase (PI3K)-related family of protein kinases [2]. Hundreds of H2AX molecules in the chromatin surrounding a DSB are phosphorylated on this residue, which can be visualized as a distinct nuclear focus by immunofluorescence microscopy [3]. Ser139 phosphorylated H2AX molecules are referred to as γH2AX. The formation of these γH2AX foci around each DSB facilitates the recruitment of DNA repair and checkpoint signaling factors [1]. There is compelling evidence that DSBs provide the primary signal for H2AX phosphorylation [1]. Accordingly, the quantification of γH2AX foci has emerged as a widely-used marker of DSBs that are detected by the cell [1,2,3,4,5,6]. After repair of DSBs, dephosphorylation of γH2AX at Ser139 has been shown to be important in allowing restart of the cell cycle and the maintenance of cell homeostasis. This critical function is largely accomplished by wild-type p53-induced phosphatase 1 (WIP1, or PPM1D), a type 2C serine/threonine phosphatase [7,8,9].

Spontaneously high levels of γH2AX foci can occur in cells without exposure to exogenous stress. Apoptotic cell death [10,11] and replicative senescence [12,13,14,15], for example, are both associated with accumulation of DSBs, and, hence, γH2AX foci formation. Somewhat surprisingly, high numbers of γH2AX foci (e.g., 50 per nucleus) have also been observed in cells within proliferating cultures of some human solid tumor-derived cell lines. Yu *et al.* [16] examined 17 cell lines chosen randomly from the NCI-60 panel of cancer cell lines and observed that cell lines expressing mutant p53 or lacking p53 function showed a significantly higher number of γH2AX foci than cell lines expressing wild-type p53. The mechanism by which wild type p53 prevents the spontaneous H2AX phosphorylation remains largely speculative.

In the current study we tested the hypothesis that endogenous H2AX phosphorylation might be associated with constitutively low expression of p21^WAF1^ (p21) and/or WIP1, both of which are transcriptionally activated by p53 and play major roles in the ATM-p53 pathway and maintenance of genomic stability (reviewed in [17]). The p21 protein was discovered in the early 1990’s and was classified as a member of the CIP/KIP family of the cyclin-dependent kinase (CDK) inhibitors [18,19]. It binds to and inhibits the activity of cyclin/CDK complexes (e.g., CDK1, 2 and 4), thus effectively blocking cell cycle progression. Subsequently, p21 was described as a multifunctional, broad-acting protein with key roles not only in cell cycle regulation but also in DNA repair, transcription, apoptosis and senescence (reviewed in [17,20,21,22]). WIP1, on the other hand, dephosphorylates γH2AX and other DNA damage response proteins (e.g., ATM, p53, BAX), thereby suppressing apoptosis [17,23].

In view of these properties of p21 and WIP1, we reasoned that loss or constitutively low expression of p21 in human cancer cells might lead to genomic instability (e.g., DSBs), triggering spontaneous γH2AX foci formation, and that low expression of WIP1 might contribute to the persistence of such foci. We performed three sets of experiments to test our hypotheses: (i) studies with the HCT116 colon carcinoma cell line and its p21 knockout derivative (HCT116p21−/−); (ii) studies with breast cancer cell lines that differ with respect to *TP53* status, and hence constitutive p21 and WIP1 levels; and (iii) studies with the p53 wild-type MCF7 cell line in which WIP1 or p21 was suppressed by pharmacological and siRNA approaches. We demonstrate that high numbers of endogenous γH2AX foci correlate inversely with expression of both p21 and WIP1, and that these endogenous foci are not associated with cells undergoing apoptosis. Aside from providing a molecular basis for spontaneous γH2AX foci, our studies suggest that p21-deficiency (absence or constitutively low expression) in human solid tumor-derived cells might provide a selective pressure for the emergence of apoptosis-resistant progeny exhibiting genomic instability.

## 2. Results and Discussion

### 2.1. p21 Loss in HCT116 Cells Promotes Spontaneous Activation of a DNA Damage Response Pathway

The HCT116 colon carcinoma cell line expresses wild-type p53 and p21 proteins and responds to moderate doses of DNA-damaging agents by predominantly undergoing premature senescence [24,25,26,27]. The p21 protein is transcriptionally activated by p53 and contributes to the control of cell cycle checkpoints, DNA repair, transcription, apoptosis, and premature senescence [17,20,21,22]. In addition, studies with the parental HCT116 cell line and its p21 knockout derivative (HCT116p21−/−) have suggested a requirement of p21 in the negative regulation of p53 protein stability [28,29,30,31,32]. Endogenous p53 in HCT116p21−/− cells showed higher transcriptional activity [32] and phosphorylation at serines associated with p53 stability and nuclear localization [24,31,32] as compared to endogenous p53 in parental cells. These observations led Hill *et al.* [31] to conclude that HCT116p21−/− cells display a classical “stressed” phenotype. Consistent with this notion, these authors observed significantly higher levels of γH2AX in HCT116p21−/− cells than in parental cells when evaluated by immunoblot analysis. Whether high levels of γH2AX in HCT116p21−/− cells reflects nuclear foci was not reported.

**Figure 1:**
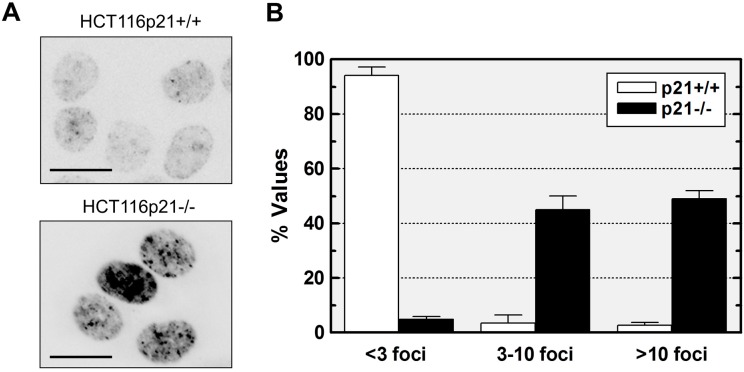
Influence of p21 loss on endogenous γH2AX foci in HCT116 cells. (**A**) Representative immunofluorescence images showing constitutive γH2AX foci in HCT116p21+/+ and HCT116p21−/− cultures; scale bar, 10 µm; (**B**) Comparison of these cultures for the proportion of cells with low/background (<3/nucleus), moderate (3–10/nucleus) and high (>10/nucleus) levels of foci. Bars, standard error (SE).

We performed immunofluorescence staining with an antibody specific for the phosphorylation of Ser139 at the *C*-terminal region of H2AX and observed a remarkable difference between HCT116p21−/− and parental cultures in terms of spontaneous γH2AX foci. Representative immunofluorescence images are shown in Figure 1A, and averaged data from multiple experiments are presented in Figure 1B. For ease of comparison, cells were divided into three categories: cells with low numbers of foci (less than 3 per nucleus, as observed with normal human fibroblasts before exposure to genotoxic agents [4,33,34]), cells with moderate numbers of foci (between 3 and 10 per nucleus), and cells with high numbers of foci (greater than 10 per nucleus). Approximately 5% and 95% of the cells within HCT116p21−/− and parental cultures exhibited low (background) numbers of foci, respectively, whereas the majority (~95%) of cells within HCT116p21−/− cultures and only ~5% of cells within parental cultures contained moderate or high numbers of foci (Figure 1B).

**Figure 2:**
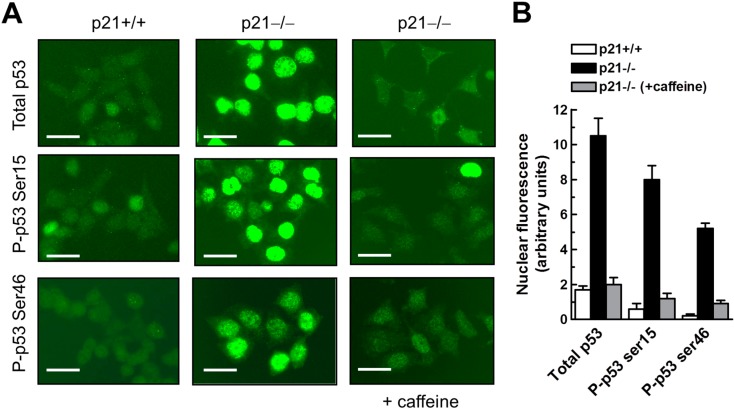
Immunofluorescence analysis of p53 protein levels and phosphorylation status in HCT116p21+/+ and HCT116p21−/− cultures before and after incubation with caffeine (4 mM; 24 h). (**A**) Representative images showing phosphorylation and nuclear localization of p53 in HCT116p21−/− cultures and suppression of these effects by caffeine; scale bar, 20 µm; (**B**) Densitometric evaluation of intensities of nuclear fluorescence staining. Bars, SE.

Given that the kinases (e.g., ATM) that phosphorylate H2AX in response to DSBs also typically phosphorylate p53, the presence of γH2AX foci in the majority of HCT116p21−/− cells suggests that these cells may also exhibit phosphorylation and nuclear accumulation of p53 in the absence of an exogenous DSB stimulus. Unfortunately the results in the literature on the influence of p21 loss on subcellular localization of p53 have been contradictory. Hill *et al.* [31] observed high levels of p53 in the nuclear fraction of HCT116p21−/− cells, whereas Peng *et al.* [35] reported the cytoplasmic sequestration of p53 in these cells. We therefore performed p53 immunostaining experiments to determine what proportion of cells within HCT116p21−/− cultures exhibit nuclear and/or cytoplasmic localization of p53. A small proportion (<5%) of HCT116p21−/− cells exhibited either cytoplasmic localization of p53 or did not express p53 at levels detectable by this assay (data not shown). The majority (>95%) of HCT116p21−/− cells, however, exhibited high levels of p53 in the absence of irradiation or other exogenous stress, and these p53 molecules were phosphorylated (e.g., at serines 15 and 46) and localized in the nucleus (Figure 2). Incubation of HCT116p21−/− cultures for 24 h with caffeine, an inhibitor of PI3K-like protein kinases [31], resulted in a marked decrease in the spontaneous phosphorylation and nuclear levels of p53 (Figure 2). Similar treatments with the PI3K-like protein kinase inhibitors wortmannin (10 μM) and LY294002 (20 μM) also suppressed nuclear p53 in HCT116p21−/− cells, although to a lesser extent than that seen with caffeine (data not shown). Unlike HCT116p21−/− cells, the majority (>80%) of parental HCT116 cells exhibited low levels and phosphorylation of constitutive p53 protein (Figure 2).

We conclude that loss of p21 in HCT116 cells promotes spontaneous activation of a DNA damage response pathway as evidenced by formation of γH2AX foci coupled with phosphorylation and nuclear accumulation of p53.

### 2.2. Loss of p21 in HCT116 Cells Is Associated with Accumulation of DNA Double-Strand Breaks (DSBs)

**Figure 3:**
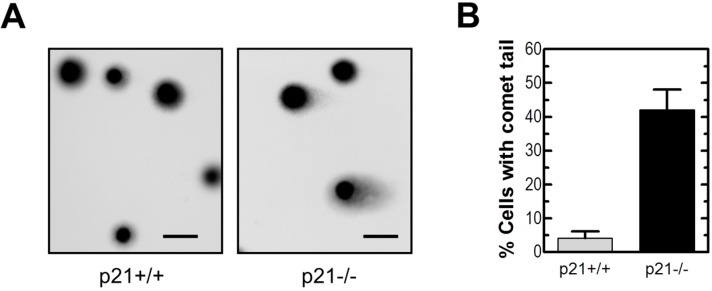
Neutral comet analysis of DSBs in HCT116p21+/+ and HCT116p21−/− cultures. Representative images (**A**) together with percentages of cells with DSBs (**B**) are presented. The mean (± SE) of the values obtained for four comet slides are shown in panel (**B**); at least 50 cells were analyzed for each slide. Scale bar, 10 µm.

The presence of cells with high numbers of γH2AX foci within HCT116p21−/− cultures suggests the spontaneous accumulation of DNA DSBs. We used the neutral comet assay to further test this notion. In this assay, the appearance of a comet tail is presumed to reflect the presence of DSBs [36]. Representative comet images and measurement of the percentages of cells with a comet tail are shown in Figure 3. Exponentially growing HCT116p21−/− cultures contained a much higher (~8-fold) proportion of cells with DSBs than the parental cultures. However, it is important to note that the proportion of cells with DSBs (~40%) in HCT116p21−/− cultures was less than that of cells with >3 γH2AX foci (~95%).

### 2.3. Endogenous γH2AX Foci and DSBs in HCT116p21−/− Cells Are not Associated with Apoptosis

**Figure 4:**
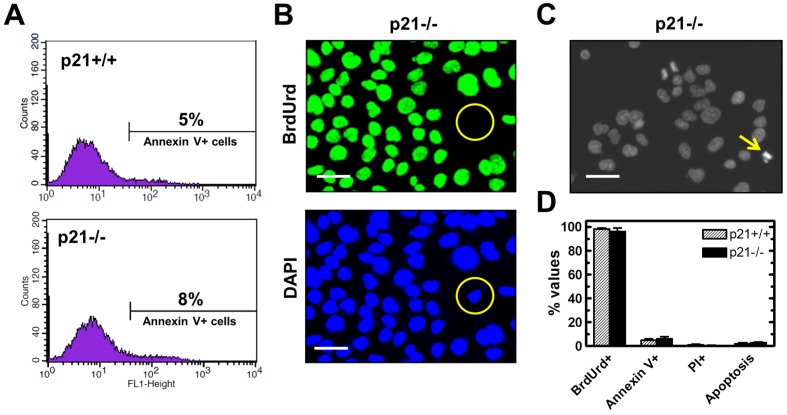
Comparison of HCT116p21+/+ and HCT116p21−/− cultures for apoptosis, viability and proliferation. (**A**) Flow cytometric evaluation of Annexin V-positive (apoptotic) cells; (**B**) Evaluation of replicating cells by the BrdUrd immunofluorescence assay. Circles mark a cell that did not exhibit DNA synthesis during the 24-h incubation period with BrdUrd; Scale bar, 20 µm; (**C**) Representative image of Hoechst 33258-stained HCT116p21−/− cells. The arrow marks a cell with apoptotic morphology; Scale bar, 20 µm; (**D**) Comparison of these cultures for the indicated parameters. Bars, SE. PI, propidium iodide.

DNA DSBs can cause loss of colony-forming ability of different human cell types without triggering apoptosis. Studies with human fibroblast strains [37] and lung carcinoma cell lines [6] have shown that DSB-induced loss of clonogenicity predominantly reflects premature senescence, and that this response correlates with sustained upregulation of p21 in p53 wild-type cells and of p16^INK4a^ (hereafter p16) in p53 mutated cells. The finding that DSBs are virtually non-apoptogenic in the fibroblast strains and cancer cell lines used in those studies is not surprising, given that both p21 [21] and p16 [38] are well known to suppress apoptosis. The HCT116 colon carcinoma cells, however, do not express p16 due to the presence of a frameshift mutation in one allele of the *INK4A* gene and hypermethylation of the promoter of the other allele [39]. Accordingly, loss of p21 function in these cells might be expected to render them highly sensitive to DSB-induced apoptosis. To test this possibility, exponentially growing HCT116p21−/− and parental cultures were evaluated by the Annexin V/flow cytometry, propidium iodide-exclusion, Hoechst 33258 staining, and BrdUrd immunofluorescence assays. The BrdUrd immunofluorescence assay was performed with cultures that were incubated for 24 h in the presence of BrdUrd (0.1 mM) to allow the selective incorporation of the analogue into the genome of replicating cells. The results are presented in Figure 4. Representative flow cytometric profiles and microscopic images are shown in panels A–C, and results from different experiments are averaged in panel D. There was no significant difference between HCT116p21−/− and parental cultures in terms of the proportion of Annexin V-positive (apoptotic) cells, propidium iodide-positive (nonviable) cells, and BrdUrd-positive (replicating) cells. Consistent with these observations, microscopic evaluation following Hoechst staining revealed the presence of <2% of cells with apoptotic morphology in both cultures.

These observations are compatible with a model in which the sustained absence of p21 function in p16-deficient cancers might create a strong selective pressure for the enrichment of cells exhibiting genomic instability coupled with resistance toward DSB-induced apoptosis.

### 2.4. Spontaneous γH2AX Foci in Mutant p53-Expressing Cell Lines in Relation to DSBs and Apoptosis

**Figure 5:**
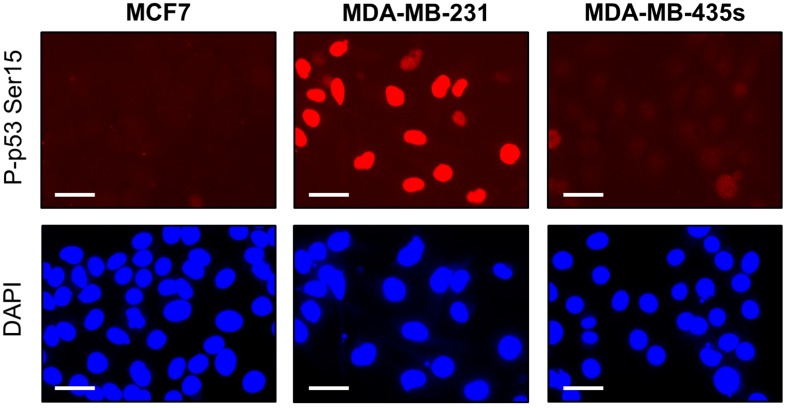
Serine 15-phosphorylated p53 protein levels in the indicated cancer cell lines. Scale bar, 20 µm.

We next compared the breast cancer cell lines MCF7, MDA-MB-231 and MDA-MB-435s for γH2AX foci, DSBs, proliferation and apoptosis. MCF7 cells express wild-type p53, whereas MDA-MB-231 and MDA-MB-435s cells express mutant p53 [21,40], which is localized in the nucleus [21]; mutant p53 in MDA-MD-231 cells is phosphorylated on serine 15 in the absence of exogenous stress ([41,42] and Figure 5).

**Figure 6:**
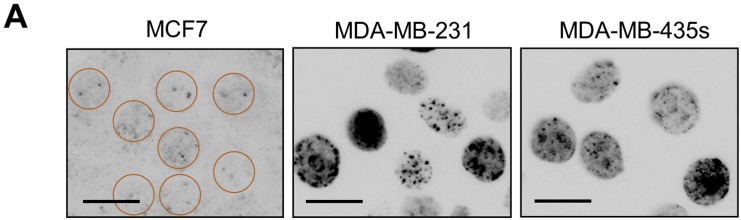

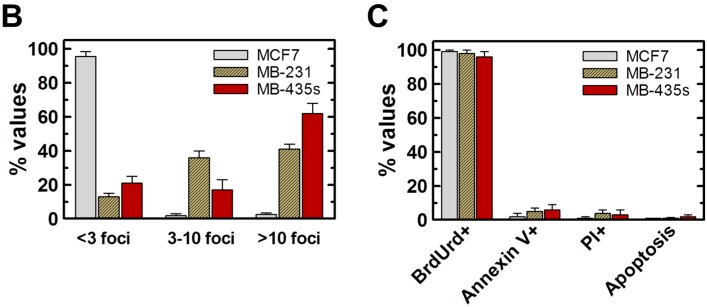
Spontaneous levels of γH2AX foci in the indicated breast cancer cell lines. (**A**) Immunofluorescence images showing constitutive γH2AX foci. Circles mark nuclei with low levels of foci; Scale bar, 10 µm; (**B**) Comparison of these cell lines for the proportion of cells exhibiting the indicated numbers of foci per nucleus. Bars, SE; (**C**) Comparison of these cultures for proliferation (BrdUrd incorporation), loss of viability (propidium iodide uptake) and apoptosis (Annexin V positive; apoptotic morphology). Bars, SE.

Immunofluorescence microscopy demonstrated the presence of moderate levels (between 3 and 10 per nucleus) or high levels (>10 per nucleus) of γH2AX foci in the majority (>80%) of cells within MDA-MB-231 and MDA-MB-435s cultures, but in only ~5% of cells within MCF7 cultures (Figure 6). The neutral comet assay also showed a higher proportion of cells with DSBs in MDA-MB-231 (~20%) and MDA-MB-435s (~30%) cultures than in MCF7 cultures (<5%) (data not shown). On the other hand, exponentially growing cultures of these three cell lines contained a similar proportion of apoptotic cells (<2%) and proliferating cells (>90%) (Figure 6C), indicating that spontaneous γH2AX foci and DSBs in the MDA-MB-231 and MDA-MB-435s cell lines are not associated with apoptosis.

### 2.5. Inverse Correlation between γH2AX Foci and WIP1 Expression

**Figure 7:**
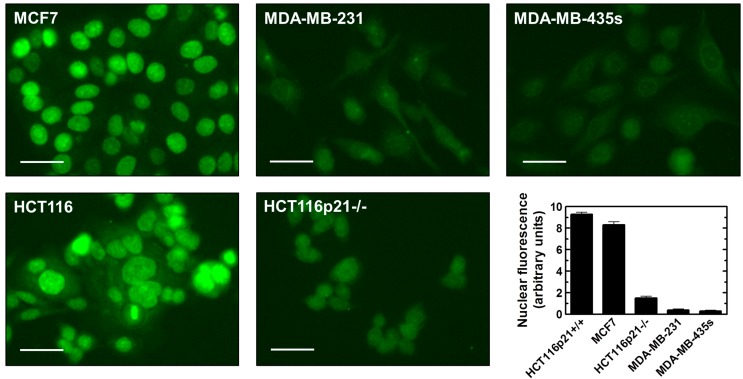
Immunofluorescence analysis of constitutive WIP1 protein levels in the indicated cell lines. Representative images together with densitometric evaluation of intensities of nuclear fluorescence staining are presented. Bars, SE. Scale bar, 20 µm.

**Figure 8:**
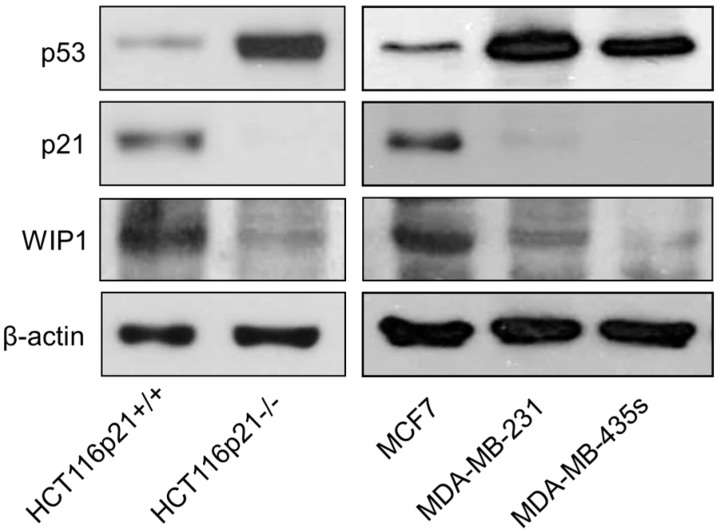
Immunoblot analysis of constitutive p53 (global), p21 and WIP1 protein levels in the indicated cell lines.

Yu *et al.* [16] proposed that high endogenous levels of γH2AX foci in some cancer cell lines lacking wild-type p53 function might reflect chromatin instability, broadly defined. This conclusion was based on the observation that high numbers of γH2AX foci in the SW756 cervical carcinoma cell line were not associated with spontaneous DSBs when evaluated by the neutral comet assay. In the current study, we have shown that HCT116p21−/− cultures contain ~95% of cells with spontaneous γH2AX (Figure 1) but only ~40% of cells with DSBs (Figure 3). Similarly, the proportion of cells with γH2AX in MDA-MB-231 and MDA-MB435s cultures (Figure 6) was greater than that of cells with DSBs (data not shown). These results are consistent with the conclusion of Yu *et al.* [16] that factors in addition to DSBs might contribute to high endogenous levels of γH2AX foci in some cancer cell lines. We focused on WIP1, which is known to dephosphorylate γH2AX in response to ionizing radiation after DSBs are rejoined [9,17]. The results of immunofluorescence and immunoblotting experiments are presented in Figure 7 and Figure 8, respectively. HCT116 and MCF7 cell lines that exhibit low (background) levels of γH2AX foci expressed detectable levels of constitutive WIP1, which was localized in the nucleus, whereas the three cell lines with high γH2AX foci levels (HCT116p21−/−, MDA-MB-231, and MDA-MB-435s) exhibited relatively low levels of WIP1.

We extended these studies to other cancer cell lines that express wild-type p53 (A549; SKNSH), mutant p53 (SUM159-PT; MDA-MB-648; BT20, SKBR3), or no p53 (SKOV3; HCT116 p53 knockout). Consistent with the observations reported above, wild-type p53-expressing cell lines contained low levels of γH2AX foci whereas cell lines lacking wild-type p53 function contained high levels of γH2AX foci (data not shown). Furthermore, levels of γH2AX foci in these cell lines correlated inversely with endogenous expression of both p21 and WIP1 (data not shown).

### 2.6. Influence of WIP1 Knockdown on γH2AX Foci

**Figure 9:**
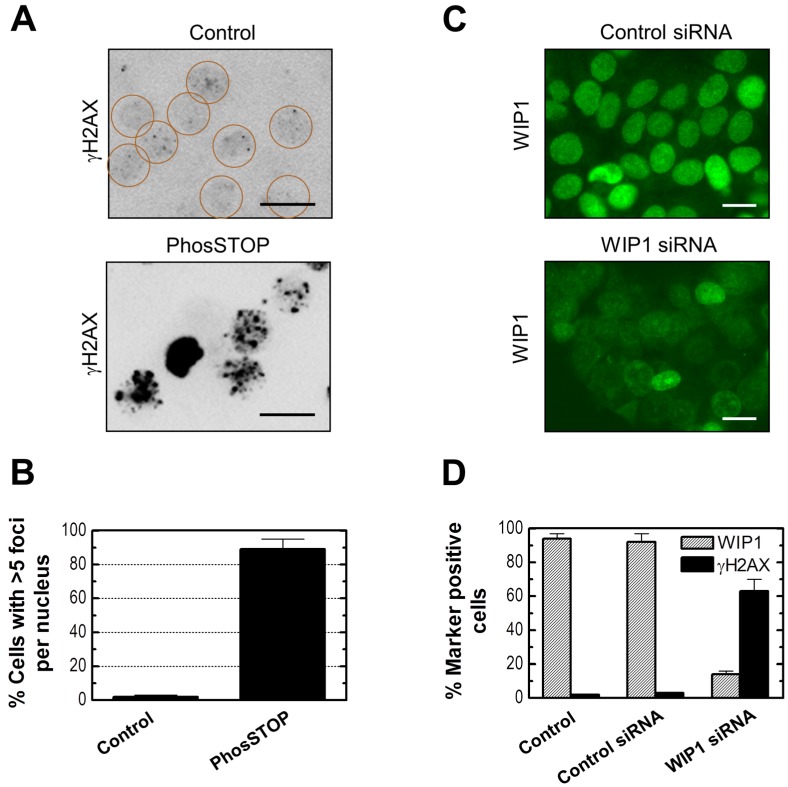
Influence of phosphatase inhibition by PhosSTOP or WIP1 siRNA on γH2AX foci in MCF7 cells. (**A**) Representative images showing presence of γH2AX foci after PhosSTOP treatment. Circles mark cells with low (background) levels of foci; Scale bar, 10 µm; (**B**) Proportion of cells with >5 γH2AX foci/nucleus for the indicated treatments. Bars, SE; (**C**) Representative images showing nuclear WIP1 levels after transfection with control siRNA or WIP1 siRNA; Scale bar, 10 µm; (**D**) Influence of transfection with the indicated siRNAs on the proportion of WIP1 positive cells (exhibiting strong nuclear staining) and γH2AX positive cells (exhibiting >5 γH2AX foci/nucleus); Bars, SE.

To determine whether phosphatase inhibition can, by itself, promote γH2AX foci formation, MCF7 cells were either treated with a phosphatase inhibitor (PhosSTOP) [43] or transfected with a WIP1 siRNA or a control siRNA [44] and then evaluated by γH2AX immunofluorescence. A 2-h treatment with PhosSTOP followed by incubation in fresh medium for 24 h resulted in γH2AX foci in the majority of the cells (Figure 9A,B). Similarly, transfection with WIP1 siRNA (50 nM) for 48 h resulted in lowering of nuclear WIP1 levels accompanied by γH2AX foci in the majority of the cells (Figure 9C,D). On the other hand, transfection with control siRNA had no influence on WIP1 levels and did not trigger γH2AX foci (Figure 9C,D).

We also determined the effect of phosphatase inhibition on DSBs and cytotoxicity (data not shown). PhosSTOP treatment or transfection with WIP1 siRNA induced DSBs in only <5% of the cells when evaluated by the neutral comet assay. In addition, under these conditions we observed marginal (<5%) cytotoxicity when assessed by microscopic examination and immunofluorescence analysis after propidium iodide staining (loss of viability) and Hoechst 33258 staining (apoptotic morphology).

### 2.7. Influence of p21 Inhibition on γH2AX Foci and Nuclear Levels of WIP1 and p53

To determine whether p21 inhibition can promote γH2AX foci, we performed studies with MCF7 cells that were either treated with a pharmacological inhibitor of p21 (UC2288 [45] or high concentrations of the CDK/cyclin inhibitor roscovitine [46]), or were transfected with a p21 siRNA [47]. In contrast to WIP1 inhibition, p21 inhibition under conditions typically used in published studies with other cell types resulted in significant cytotoxicity in MCF7 cultures. For example, a 48-h transfection with 50 nM p21 siRNA resulted in extensive cell detachment, whereas transfection with WIP1 siRNA under similar conditions elicited marginal (if any) effect (data not shown). Immunofluorescence analysis after propidium iodide and Hoechst 33258 staining indicated that virtually all detached cells had lost viability and a high proportion (~40%) exhibited apoptotic morphology (nuclear condensation and fragmentation) (data not shown).

**Figure 10:**
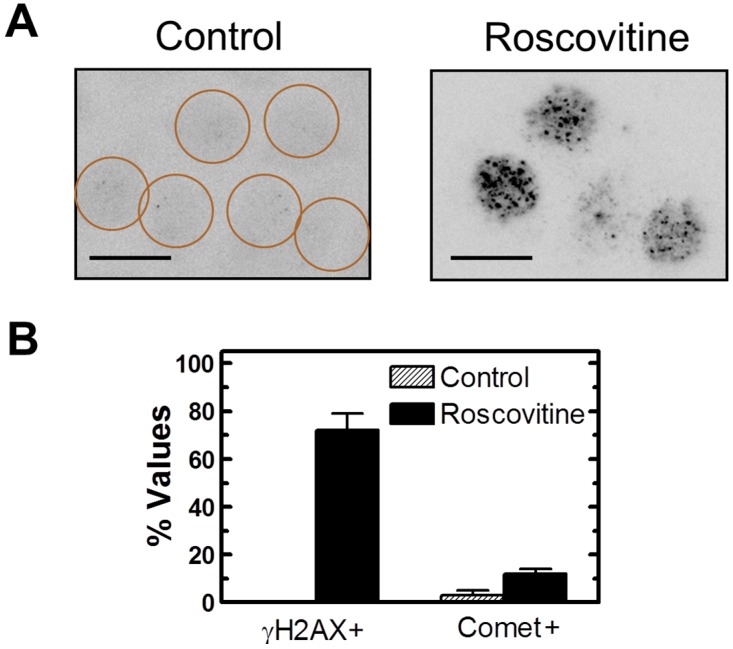
(**A**) Representative images showing the presence of γH2AX foci in MCF7 cells treated with roscovitine (40 µM) for 24 h. Circles mark cells with low (background) levels of foci; Scale bar, 10 µm; (**B**) Influence of roscovitine treatment on percentages of cells with γH2AX foci (exhibiting >5 foci/nucleus) and DSBs (exhibiting ≥10% fluorescence in comet tails). Bars, SE.

We observed γH2AX foci in the majority (~70%) of MCF7 cells that remained adherent after treatment with roscovitine (Figure 10), treatment with UC2288 (data not shown), or transfection with p21 siRNA (data not shown). On the other hand, neutral comet assay demonstrated the presence of DSBs in only ~10% of these cells (Figure 10B and data not shown).

**Figure 11:**
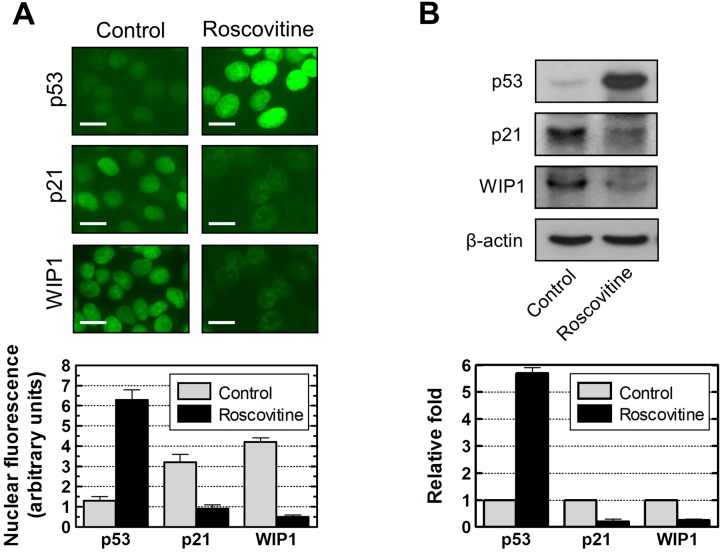
Influence of p21 downregulation by roscovitine (40 µM, 24 h) on p53 and WIP1 protein levels in MCF7 cells. (**A**) Immunofluorescence analysis of nuclear protein levels for control and roscovitine-treated cultures. The histograms for the indicated proteins were obtained from densitometric analysis of intensities of nuclear fluorescence staining from at least four coverslips; over 100 cells were evaluated for each coverslip. Bars, SE; Scale bar, 10 µm; (**B**) Immunoblot analysis of global protein levels before and after roscovitine treatment. The histograms for each protein were obtained from densitometric analysis of the band, normalized to actin, from at least three independent immunoblots. Bars, SE.

Roscovitine at 40 µM was less toxic than p21 siRNA and UC2288 when used under conditions required to diminish constitutive p21 protein levels in MCF7 cells. We, therefore, used roscovitine to determine the effect of p21 suppression on WIP1 and p53 protein levels. If p21 functions as a positive regulator of WIP1, as suggested by the studies reported above, then suppressing p21 should also lead to suppression of WIP1 (a negative regulator of p53) accompanied by upregulation of p53. The results of immunostaining and immunoblotting experiments are presented in Figure 11. Roscovitine treatment of MCF7 cells caused downregulation of both p21 and WIP1, but led to upregulation and nuclear localization of p53. We conclude that p21 does function as a positive regulator of WIP1 in this breast carcinoma cell line.

## 3. Experimental Section

### 3.1. Cells and Cell Culture

The human colon carcinoma cell line HCT116, which is known to activate the p53-p21 axis after DNA damage, and a p21 homozygous knockout derivative of this cell line (HCT116p21−/−) were generous gifts of Dr. Bert Vogelstein (Johns Hopkins University, Baltimore, MD, USA). All other cancer cell lines used in the present study were purchased from the American Type Culture Collection (Rockville, MD, USA). Cells were cultured as monolayers in DMEM/F12 nutrient medium supplemented with 10% (*v*/*v*) fetal bovine serum as described [25]. All cultures were free of *Mycoplasma* contamination.

### 3.2. Antibodies

Antibodies to p53 (DO-1), p21, WIP1, and β-actin were purchased from Santa Cruz Biotechnology (Santa Cruz, CA, USA). An antibody to bromodeoxyuridine (BrdUrd) was purchased from Becton Dickson (San Jose, CA, USA). A phosphospecific antibody to γH2AX (Ser139) was purchased from Cedarlane Laboratories (Hornby, ON, Canada). Phosphospecific antibodies to Ser15 and Ser46 of human p53 were purchased from Cell Signaling (Beverly, MA, USA). Alexa Fluor 488 antibodies (goat anti-mouse IgG and donkey anti-rabbit IgG) were purchased from Molecular Probes (Eugene, OR, USA).

### 3.3. Immunoblot and Immunofluorescence Techniques

Global levels of specific proteins were evaluated by immunoblotting as described [48]. Protein, γ-H2AX, and BrdUrd immunofluorescence assays were performed as described [49].

### 3.4. siRNA-Mediated Gene Silencing Analysis

Transfection with siRNA targeting WIP1 (D-004554-01; Thermo Scientific Dharmacon; Chicago, IL, USA), p21 (SC37007; Santa Cruz), or control siRNA (D-001210-01; Thermo Scientific) was performed using published protocols [50,51]. In a pilot experiment we compared different transfection reagents and found transfection using Lipofectamine 2000 (Invitrogen, Carlsbad, CA, USA) to yield reproducible results. Cells were incubated with the Lipofectamine 2000 transfection mixture containing siRNA sequences for 48 h and then analyzed by immunofluorescence microscopy.

### 3.5. Neutral Comet Assay

DSBs were quantified using a commercial neutral comet kit (Cell Signaling Technology, Beverly, MA, USA) as described [4]. DSB frequencies were determined from the percentage of total fluorescence in the comet tail as described [4]. Cells exhibiting ≥10% fluorescence in comet tail were scored as positive [4].

### 3.6. Annexin V/Flow Cytometry Analysis

Apoptosis was evaluated by the Annexin V/flow cytometry assay as described [49].

### 3.7. Propidium Iodide-Exclusion Assay

Cell viability was evaluated by the propidium iodide (PI)-exclusion assay as described [37].

## 4. Conclusions

High levels of spontaneous γH2AX foci that arise in certain human cancer cell lines have been postulated to reflect DNA replication stress, telomere dysfunction, and chromatin instability [13,16,52,53,54,55]. In this report, we have demonstrated an inverse relationship between γH2AX foci and expression of both p21 and WIP1 in proliferating cultures of the panel of cancer cell lines that we examined. Our findings are consistent with the known properties of p21 and WIP1. In addition to its pivotal role in activating cell cycle checkpoints, downregulating apoptosis and inducing premature senescence, the p21 protein is recruited by γH2AX to DSB sites in genomic DNA [56,57,58,59] and promotes the replication-coupled repair of DSBs [59]. Loss or constitutively low expression of p21 would result in diminished ability to effect replication-coupled DSB rejoining as compared to wild-type cells expressing “normal” levels of p21, and low expression of WIP1 would result in γH2AX foci persisting for long times after rejoining of DSBs, which would be visualized as spontaneous γH2AX foci. In support of this model, down-regulating/inhibiting either WIP1 or p21 in MCF7 cells resulted in accumulation of γH2AX foci in the absence of exogenous stress.

Induction of WIP1 in response to genotoxic stress is mediated by p53-dependent and -independent mechanisms in human cancer cells [44]. Our current results suggest that p21 might function as a positive regulator of WIP1 independent of wild-type p53 activity. This conclusion is based on the findings that: (i) p21 knockout HCT116 cells express constitutively low levels of WIP1 despite the spontaneous phosphorylation and nuclear accumulation of (wild-type) p53 when compared to parental cells; and (ii) down-regulating p21 in MCF7 cells results in low expression of WIP1 but nuclear accumulation of (wild-type) p53. Given that WIP1 is a key negative regulator of p53 [17,60,61] and apoptotic cell death [23], our results suggest that one mechanism by which p21 might exert its inhibitory effects on p53 and apoptosis might be through regulating WIP1, at least in these widely used cancer cell lines.

**Figure 12:**
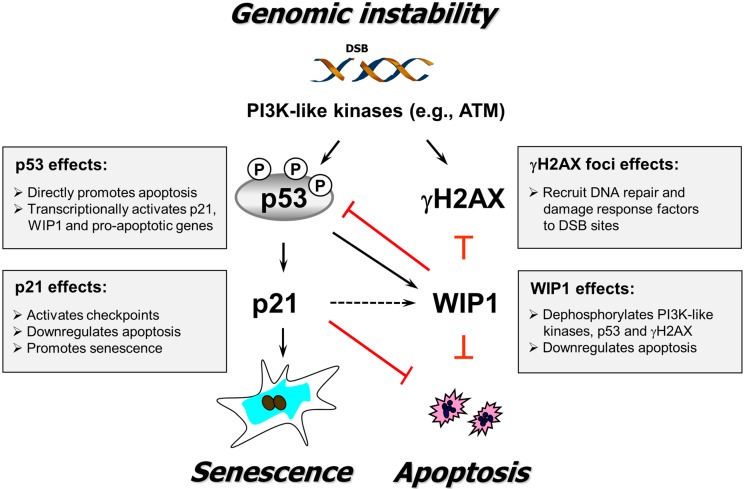
A schematic of the relationship between p53 signaling, γH2AX foci, DSB repair, and biological outcomes triggered by genotoxic stress (e.g., spontaneous DSBs) supported by the current study. Solid arrows indicate stimulation and T-shaped lines indicate inhibition. The dotted arrow indicates positive regulation of WIP1 by p21 suggested by studies reported herein. Some known functions of p53, p21, WIP1, and γH2AX are indicated (for details, consult [17]).

As illustrated in Figure 12, our findings suggest a novel role for p21 in the response of solid tumor-derived cell lines to genotoxic stress (e.g., spontaneous DSBs); namely, its ability to form a regulatory loop with WIP1 and hence potentially contribute to dephosphorylation of p53, γH2AX, and probably other key participants in the DNA damage surveillance network. Thus, our findings add to the complexity of p21 functions and lend credence to the cautionary note made by us [17] and Warfel and El-Deiry [20] that a greater insight into the molecular and biological consequences associated with p21 loss is crucial to determining whether modulating p53–p21 signaling might be a promising approach for the treatment of certain types of cancers. Towards this end, further studies are warranted to determine: (i) the generality of our observations (*i.e.*, interplay between p53, p21 and WIP1) in different types of non-transformed and cancerous cells; and (ii) whether p21-mediated WIP1 regulation occurs at the transcriptional, post-transcriptional, or post-translational level.
